# Co-designing animated videos to explain large language models and their use in healthcare and research

**DOI:** 10.3389/fdgth.2026.1758463

**Published:** 2026-04-13

**Authors:** Elizabeth Remfry, Jaya Chaturvedi, Sarah Markham, Elizabeth Ford, Mel Ramasawmy

**Affiliations:** 1 Queen Mary University of London, London, United Kingdom; 2 King’s College London, London, United Kingdom; 3 Brighton and Sussex Medical School, Brighton, United Kingdom

**Keywords:** AI literacy, co-design, large language models, LLMs, patient and public involvement and engagement, PPIE

## Abstract

**Introduction:**

The increasing development of large language models (LLM) in healthcare research is taking place without patient and public involvement and engagement (PPIE). Part of the challenge is the lack of accessible educational resources to promote literacy around LLMs.

**Methods:**

We employed a co-design approach with 6 PPIE contributors from Tower Hamlets, London to develop educational animations about LLMs. We conducted 7 pilot sessions that included hands-on interactive activities to develop scripts and storyboards for the animations. Animations were external validated by additional PPIE groups and experts before professional animation production.

**Results:**

Two accessible 2-minute animated videos were successfully co-designed in English and Bengali. The first explains LLMs in the context of daily life and the second on LLMs in healthcare research. Both animations use a factory analogy to describe LLMs functionality while avoiding anthropomorphisation that could mislead audiences about LLMs inner workings and capabilities. Both animations are freely available for reuse.

**Discussion:**

The co-design process allowed us to prioritise diverse PPIE voices throughout development, ensuring that animations are appropriate for a wide audience. Challenges included incorporating sometimes conflicting perspectives and achieving balanced portrayals of LLM benefits and risks. We hope these animations will support PPIE contributors to have a say in how LLMs are used in healthcare and research.

## Introduction

The use of large language models (LLMs) in healthcare is expanding rapidly, driven by LLMs’ ability to handle large volumes of diverse text. Healthcare generates vast amounts of text data, ranging from clinical notes to administrative letters (1), which has led to an excess of applications of LLMs. For example, healthcare professionals are currently using LLMs for writing and transcribing clinical notes (2), LLMs are being used in research to develop clinical decision support tools using data extracted from unstructured clinical text and patients are using LLM-based tools to ask health related questions (3).

Although there has been an increase in the development and use of AI-based systems in healthcare, these are often not designed in collaboration with patients. In parallel, there is a growing emphasis on the involvement of patients and the public in healthcare research and participatory approaches to artificial intelligence (AI) (4–6). However, engaging with patients around AI has been held back by many of the existing challenges with public and patient involvement and engagement (PPIE) work, such as limited resources and funding, and a lack of training for communicating complex topics to lay partners (7). Additionally for LLM research, the challenges also mirror those seen in statistical methodology research in healthcare, where there is a perception that some technical knowledge is required to contribute and hesitation from researchers who often feel unprepared to explain technical aspects of their research (8, 9).

One of the challenges for PPIE applied to LLM research is ensuring that we are able to communicate about AI and LLMs as transparently as possible (10). This is needed so that PPIE contributors can meaningfully contribute their lived experience expertise to research decisions made during AI development and deployment. AI literacy is not a prerequisite for PPIE contributors to participate in AI research, but it is a requirement for researchers to provide this context so that PPIE contributors can make informed decisions (10). Typically in AI research, background information is provided to participants about AI technologies, however providing information that is easily understood by participants has been flagged as a major challenge (11). Although there are resources to explain key AI concepts, such as infographics and animations produced by Understanding Patient Data that explains the use of large volume healthcare data (12), Google's Teachable Machine which explores classifiers (13) and educational animations about algorithms (14), there are limited resources on LLMs aimed at a lay audience.

This gap is amplified by the low levels of AI literacy in the UK, with around 73% of adults reporting no AI training or education (15) and AI literacy is not yet on the national school curriculum. Not only may this discourage people from getting involved with research involving LLMs, it can also have major consequences for the general public, with recent high profile cases involving individuals who have asked ChatGPT for health advice resulting in serious negative outcomes (16). Although technology is moving particularly fast, with private companies such as OpenAI marketing clinical LLM tools as ’solutions for healthcare’ (17), this rapid expansion has not been matched with the development of critical AI literacy among users. This is further compounded by the prevalent narrative and hype about LLMs which have been described as ‘magical’, ‘god-like’ (18) or ‘sentient’ (19), which can inflate expectations and obscure immediate concerns about safety and appropriate use (20).

Co-design offers a potential approach to design appropriate information to support PPIE work in AI research. Co-design has a rich history in healthcare service design and can incorporate different stakeholder's perspectives, such as patients, clinicians, researchers and healthcare professionals (21). This approach recognises the differing forms of knowledge and perspectives individuals bring to the design process and places equal importance on lived experience and technical knowledge (22) which is important when we want to ensure that materials are accessible as well as technically accurate. Previous research has used co-design to develop health information materials (23), and has highlighted how this approach can also help facilitate accessibility of knowledge by simplifying complex concepts (22), which is key in AI information provision.

This paper describes the process of co-designing two animated videos to explain LLMs and their use in healthcare and research to patients and the public. Through a series of collaborative workshops with PPIE contributors and researchers, we developed two animations that help explain how LLMs work, how they are used in our daily lives and their application in healthcare and research. These resources were designed to support PPIE members to make informed and effective contributions to research using LLMs.

## Materials and methods

### Research team

Six PPIE contributors from Tower Hamlets were invited to participate in this project. All PPIE contributors were recruited by Social Action for Health (SAfH), our community partner (24) using purposive sampling. SAfH is a community-based charity in East London that provides services and support to people most affected by health inequalities and recruited PPIE contributors through their existing community projects.

The group of PPIE contributors reflected the demographics of Tower Hamlets: half the group were female, ages ranged from 40 to 70 years, 50% self-identified as British Bangladeshi and contributors came from diverse socio-economic backgrounds. All the contributors had heard of AI, however many contributors had no previous experience of using or interacting with LLMs or had never heard of LLMs or commercial names such as ChatGPT. The experiences that the contributors had previously had with ChatGPT or other LLM-based tools came from personal use, hearing about it through their children, or through the media. All PPIE contributors were reimbursed for their time.

Alongside the PPIE contributors were a team of five academic researchers and an experienced PPIE representative. The academics included a PhD student, two post-doctoral research associates and an associate professor. Researchers were from three different UK institutions, Queen Mary University of London, Kings College London and Brighton and Sussex Medical School, and represented different academic disciplines spanning health data science, computer science and social sciences.

### Co-design workshops

**Figure 1:**
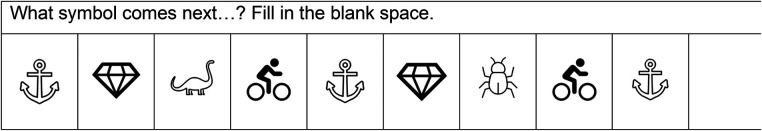
Example of the games used to mimic the probabilistic nature of LLMs.

We ran a series of 7 workshops, 2 h each, from September 2024 to January 2025 to facilitate the co-design of educational materials about LLMs. Workshops were all held in English, either at SAfH offices or on campus at Queen Mary University of London. The initial 2 workshops introduced the concept of LLMs to the PPIE contributors. This covered the wording that is typically used to talk about LLMs and provided various examples of use both in daily life and healthcare settings. PPIE contributors were invited to share with each other any existing knowledge and experiences with using ChatGPT or other commercially available LLM-based tools. To facilitate an understanding into how LLMs work on a technical level, the academic researchers designed simple games that emulated the probabilistic nature of LLMs. For example, sentence and pattern completion games, as seen in Figure 1, were used and completed as a group. These provided a level of understanding on how LLMs learn patterns and use patterns to complete sentences.

Additionally, we ran a hands-on exploration of commercial products like ChatGPT (25). In groups of 2 or 3, PPIE contributors were given a laptop, provided with a login to ChatGPT, and were encouraged to use the LLM in any way they wanted. They were also given pre-written prompts in English designed to highlight different aspects of LLMs, such as gender bias. For example, PPIE contributors were encouraged to ask ChatGPT to write a short story about a firefighter or a nurse. As a group we then discussed these stories, and why gender-typical presentations were generated by the LLM. PPIE contributors were encouraged to ask questions of the academic researchers to ensure that all relevant aspects were considered before narrowing down the scope and content of the animation.

In workshop 3 and 4, we did various brainstorming activities to understand what, as a group, we felt that the general public would want to know about LLMs, as well as what we think our friends and families should know. We particularly spent time discussing how best to represent how LLMs work in a fun and accessible way and the format of this material, whether infographic, animation, video, TikTok or other format.

We had initially planned for 4 workshops but extended this to 7 workshops after it was apparent that additional time was needed to design the images and script after animations were chosen as the best medium. For inspiration we watched various videos together to decide on the style of the animation. One researcher (MR) provided initial sketches for the storyboard based on the ideas of the PPIE contributors, which enabled the group to design the visual narrative. As the script developed these sketches were turned into a PowerPoint presentation, with images/sketches on each slide to accompany a short piece of text. Throughout all the animation design, we engaged in an iterative co-design process, whereby feedback from PPIE contributors and researchers was continually used to adapt and improve the animation.

Finally, to assist with the creation of this article, we held 1 additional workshop. Rather than asking PPIE contributors to read the draft article and provide feedback, which is a typical approach and not suitable for this research team, we held a feedback session in person. The main findings, interpretation of the co-design process and figures used in this article were discussed as a group and PPIE contributors provided feedback on the interpretation, as well as suggesting any additional concepts to be included in the written document.

### Translation

In order to make the animations accessible other communities, these were translated into Bengali. Bangali was chosen as an appropriate language given that it is the second most common language spoken in Tower Hamlets (26), where the PPIE contributors and Queen Mary University of London are located. SAfH helped to recruit two additional Bengali-English bilingual contributors to the project, who along with two of the original contributors, who were Bengali-English speakers, helped adjust the Bengali translation. Firstly, the finalised script and on screen text was translated into Bengali by a professional translation company. Secondly, over two 1-hour workshops the translation group adapted and refined the translated script to ensure that it was linguistically accessible and culturally appropriate. All translators were reimbursed for their time.

### Additional feedback

As a form of external validation, prior to the final animation being developed, the material was shared with two other PPIE groups (*n* = 18) and a group of academic experts and non-experts (*n* = 25) to collect feedback from contributors who were not involved in co-designing the material. Once an initial version of the animation had been made by the professional animation company, we also conducted a final workshop with PPIE contributors, translators and an additional 6 contributors, reflecting a wider age and educational range. Feedback from this final workshop contributed to final edits and attendees helped to select the voice over artist based on short sample audios.

### Animation development

We worked with a professional animation company, Really Bright Media (27), to convert the two scripts and storyboards into live animations based on the drawings and script developed through the co-design process.

## Results

The research team co-designed two animations on LLMs and their use in healthcare research. These animations are freely available online and for reuse: https://youtu.be/Hn6ONvJgm1w?si=0oy8mjhJyTlCP-sZ.

The first animation was focused on LLMs in our daily lives, providing alternative names and commercial names of commonly used LLMs as well as examples of how we can use them. It briefly described how they work and outlined some of the potential positives and negatives of LLM tools. The second describes LLMs in healthcare and research, it provides examples of where LLMs could be used by patients, clinicians and researchers, how they work and potential benefits and risks. Both animations are similar in style and content, with the second animation focused on providing healthcare related context.

The development of both animations involved continual design choices, several of which we highlight below. Before developing any content we explored the best communication format, discussing infographics, written materials, animations and social media content before deciding on animations. The decision reflected the research group's understanding that animated content is more visually interesting and could help to support the viewer make an informed decision about the use of LLMs. The envisioned approach would use facts, presenting both sides of the argument in a neutral way to allow the viewer to make up their own mind. A key discussion point was how to establish trust in a video. The research group decided that including recognisable logos, such as the university logo, the NHS logo or other reputable institutions could infer a level of confidence in the animation.

**Figure 2:**
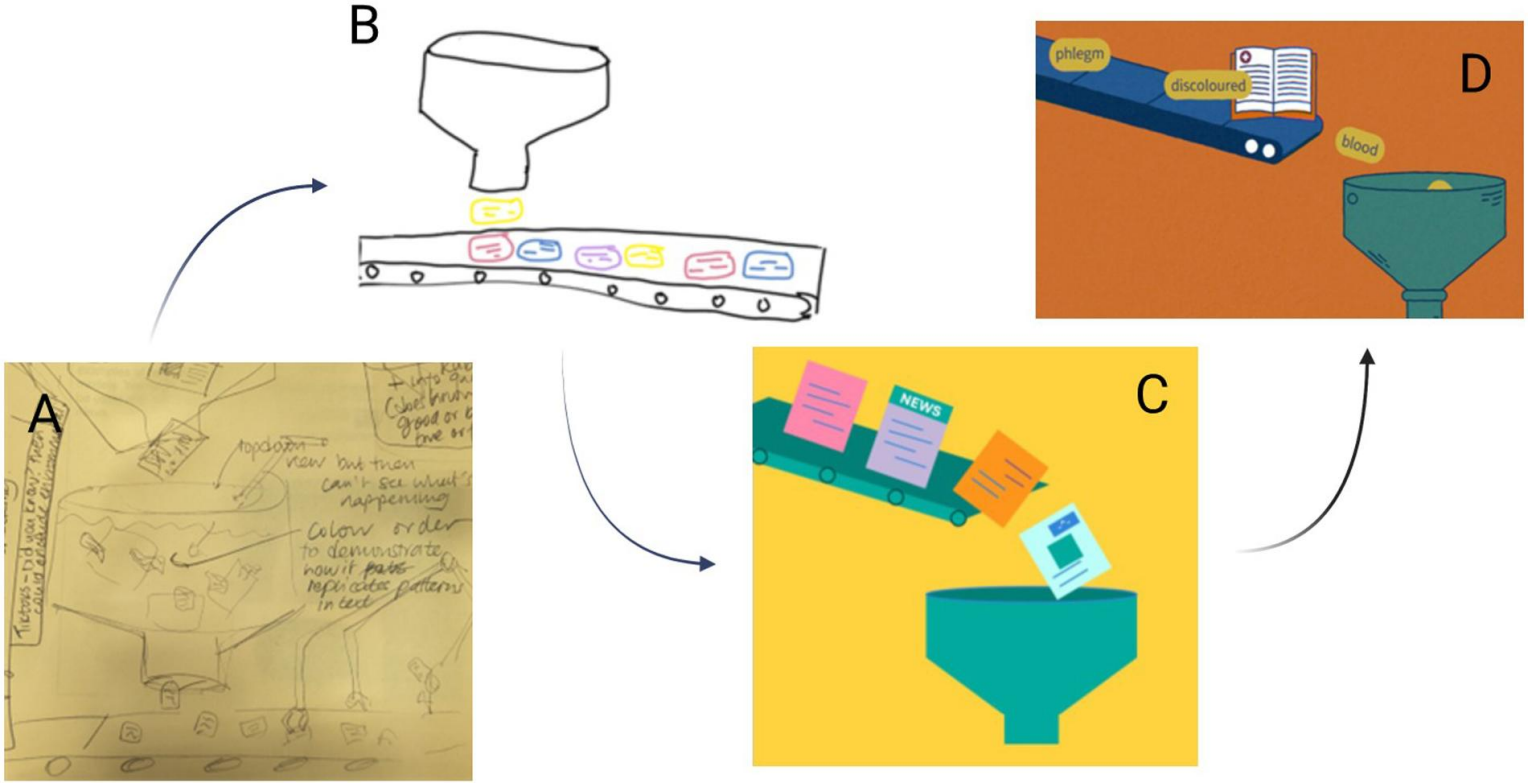
The development of the factory line, showing in **(A)** the initial pen and paper sketches, **(B)** a digital drawing, **(C)** an illustrative drawing and **(D)** the final professional version.

The factory analogy was developed through an idea of one of the PPIE contributors, who felt that the word prediction element of LLMs was key to understanding how and why errors can occur. The factory analogy frames LLMs as word factories that process and produce text in a procedural and predictable manner. This concept was talked through with the technical team members to ensure that it accurately reflected the abilities of LLMs, and the team agreed that this factory framing also avoided anthropomorphising LLMs. In Figure 2 each panel shows the development of the factory analogy over the course of the workshops, starting with a hand-drawn version which was transformed into digital drawings, PowerPoint shapes and the final animation Text can be seen moving along a conveyor belt, before being tipped into a funnel. This funnel represents the black box of an LLM, and out of the funnel words are produced and ordered in response to an input prompt. This was considered enough detail without overloading viewers with information. An example that was given by a PPIE contributor was that “*ChatGPT is like a car, I don't need to know how the car works, I just need to know how to use it*”.

We decided to use LLMs as an acronym rather than saying Large Language Models throughout the animation. However it was difficult to decide on a title of the video, given the different terms used to describe LLMs and the different brand names that people recognise. Most PPIE contributors knew of ChatGPT but had never heard of the term LLMs and used the term ‘AI’ and ‘ChatGPT’ interchangeably.

A key addition within the second animation was providing information that addressed data security concerns. As this animation was made for general use in healthcare research, rather than for a specific use case, there are various ways that LLM-based tools are employed in healthcare and these can be developed by different organisations or private companies. For example, the use of personal data is highly regulated in medical devices and academic research, however this is completely different to an individual using an LLM-based tool such as ChatGPT which can share personal data. In this case, the academic researchers in the project felt that we should highlight data protection within research, and provide links to additional information about health data protection within the UK, such as provided by Understanding Patient Data (28).

**Figure 3:**
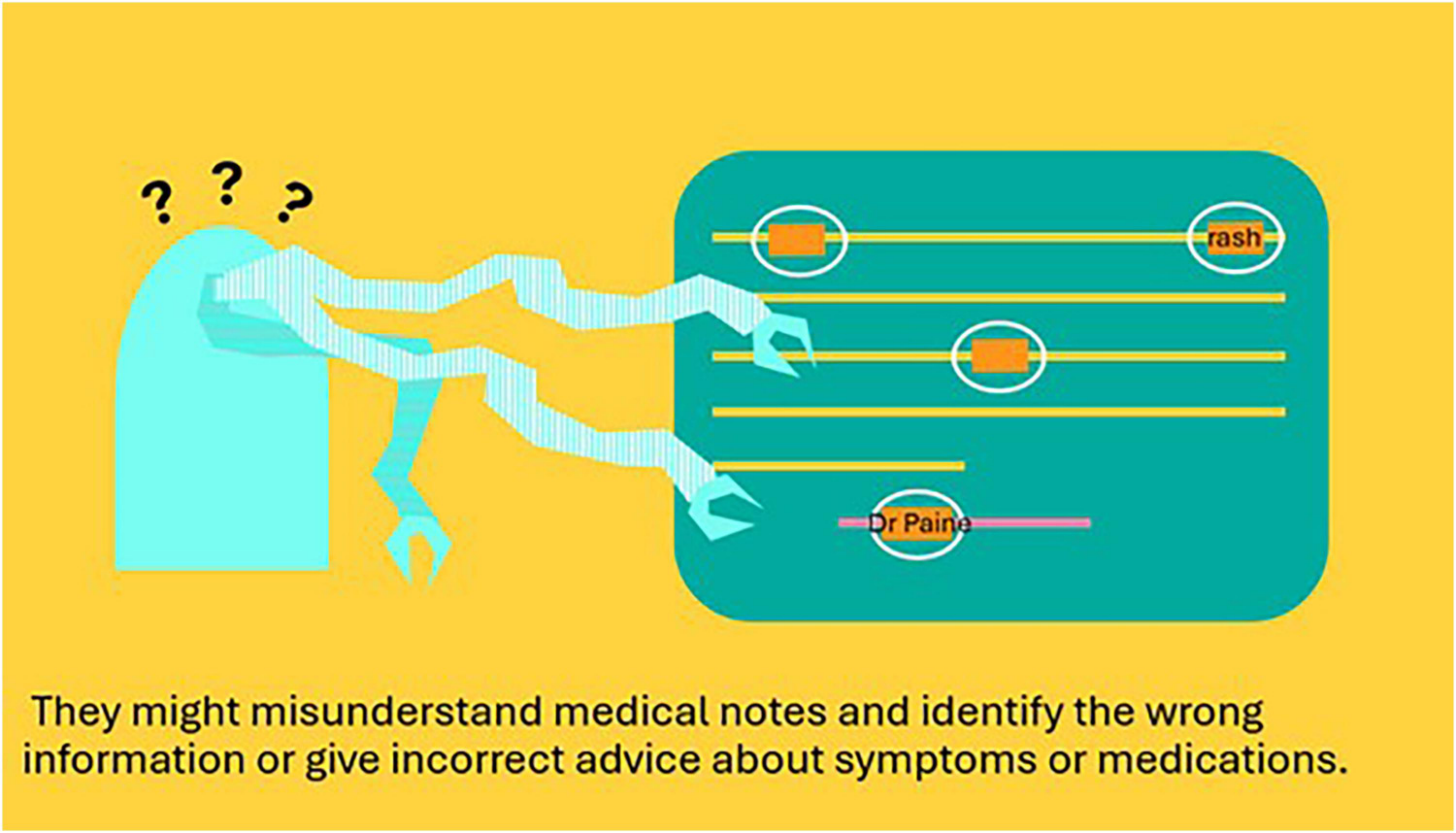
An example of animation slide mock up that highlights how an LLM system may mistake the work “Dr paine” as a pain symptom.

The animations were closely restricted by the amount of time, not only to be engaging to watch, but also to be affordable given the allocated funding. There were various other ideas ultimately deemed out of scope due to financial and time restraints. These include the PPIE contributors' recommendation that we could adapt parts of the animation into TikTok friendly videos, so that it could reach a wider audience. Additionally, the team designed part of the script to highlight how LLMs can fail to identify differences in words due to the context which can be problematic within healthcare settings. The example developed was when using an LLM-based tool to extract symptoms from a clinical note, the LLM tool may incorrectly extract a name like Dr Paine, shown in Figure 3. Additionally, we did not include any information on the environmental impact of LLM-based tools, although this was a topic of discussion throughout our workshops.

Additionally, the translation process was an important design choice. As several of the PPIE contributors spoke Bengali they felt it was important to get a range of perspectives on an appropriate translation. In Bangladesh there are around 12 dialects and 100s of different accents (29) therefore it was beneficial to recruit translators with different dialects or accents. The translator group then spent considerable time ensuring that words were understandable across as many dialects as possible.

## Discussion

This paper describes the co-design of two animated videos explaining LLMs and their use in healthcare and research. Our main objective was to co-design accessible resources that can facilitate meaningful patient and public involvement and engagement in future work. This was achieved through the co-design process that prioritised PPIE voices throughout the development of the animations, ensuring that the animations included easy to understand content with appropriate imagery allowing for accessibility without oversimplification.

Animations were chosen by the team as their preferred format for communication. This is in line with previous research that has highlighted the benefits of utilising animations to present complex health information and improve information provision (30). Projects have successfully created tools and animations to help describe statistical methods (9) and bias within data (14) for both PPIE contributors and the general public. Our work extends this approach to the rapidly evolving field of LLMs in healthcare.

In this study we found the co-design approach useful as a way to incorporate the perspectives of diverse PPIE contributors and researchers from different disciplines. The initial co-design workshops allowed the team to develop a level of shared understanding of LLMs and the iterative approach encouraged everyone to continually learn about LLMs and adjust their own perspectives in response to others' experiences. Multiple workshops provided us with time to support relationship building in the team, which has previously been highlighted as an essential foundation for trust in the co-design process (31). A PPIE contributor described “*the journey is combined, we are all part of the process, we all develop together and learn from each other”* reflecting on the process between researchers and PPIE contributors. Working as a group was also seen as beneficial by the PPIE contributors, who also said that they would not have worked on the project otherwise. The group dynamic also helped support people and amplified the importance of lived experience to the group. A PPIE contributor said: “*I don't come from a very educated background, I was nervous at first but with the support of everyone it made it easier. I didn't feel left out. I can talk about my experiences, my everyday life, my health and bring this forward to other projects*” which helps demonstrate how the perceived value of their lived experience changed through the process.

Co-design has been suggested as a method to challenge traditional power imbalances (32) particularly the long-held ones within healthcare and academia. We recognised that the process of co-developing educational materials on a technical subject contains an implicit inequality in knowledge, in which the research team are recognised to hold the expertise, and non-academic participants may minimise their own knowledge and experiences (33). Other co-development projects have highlighted the risk of the research team acting as an ‘epistemic filter’, deciding which knowledge was worth including or excluding (33). In this project we intentionally ensured that the academic team included an experienced PPIE representative and had disciplinary diversity which helped support us to consider the information needs of the group when designing the initial workshops. To work towards equalising power in this setting, we tried to create an open atmosphere that allowed PPIE contributors to take the discussions in any direction, bring their own knowledge to the table and have hands-on time with LLM-based tools to facilitate different forms of learning. This open environment and sharing of knowledge was reflected in feedback from one PPIE contributor who stated that “*no one was gatekeeping information*”.

This richness of perspectives within the research team and PPIE contributors was a strength but often led to moments of negotiation. PPIE contributors were able to identify what was useful for non-technical audiences, which at times diverged from what researchers assumed was important. For example, PPIE contributors were often focused on positive attributes of LLM-based tools, and ensuring that everyone has the skills to be able to benefit from using them, whilst academic researchers were often more negative, wanting to forefront the potential risks of using tools. To create a feeling of ownership we resolved these through discussion, and where possible prioritised PPI contributor preferences. The team worked to maintain these preferences throughout iterations and the animation process and to avoid disappointment and disengagement with the process if contributions were not implemented (34).

Translation of both animations into Bengali represented an extension of the co-design process. We aimed to take a ‘thoughtful' approach in which professional translation was iteratively tested and improved with the Bengali-speaking PPIE group to ensure readability, avoid complicated phrases and jargon, and consider cultural nuances in concepts and spoken language (35).

This co-design approach also had intrinsic benefits that extended beyond the creation of educational materials. For the majority of the PPIE contributors they had not previously been involved in research, yet due to the positive experiences of this study, several expressed an interest to be connected to future research. A key moment that highlighted a sense of co-ownership of the animations came when three PPIE contributors presented the animations at a health conference in Glasgow, and again at a launch event in London which provided a space to bring the community together and display the animation to researchers, friends and family, and members of the community. Additional benefits of PPIE work are often written about in terms of an individual's enhancement of skills and knowledge (36, 37). A PPIE contributor commented: “*It also helped to build up personal experiences and work experience”*, whilst another reflecting on talking at the symposium said “*It has opened doors, I'm more confident, it gave me something to talk about in front of a room of professionals”*. Additionally to new skills and knowledge, we also reflected as a group that the project had been enjoyable, and this has been a key reason why some PPIE contributors went on to seek out future PPIE opportunities.

### Strengths and limitations

Our work followed good practice for co-producing educational materials, such as early engagement with public contributors, a diverse PPIE contributor team, and ensuring PPIE contributors made the final decision about the design and content of the easy to understand materials (38). All of the PPIE contributors in this study were local to Tower Hamlets, and represented ethnic, age and socio-demographic status diversity. For the majority of PPIE contributors this was their first involvement in PPIE work. This is in contrast to the majority of PPIE researchers in healthcare, who are typically experienced in PPIE, white, retired and middle class (39). Additionally as animations are also available in Bengali, we hope that this will support other PPIE contributors to get involved in AI research, particularly in ethnic minority groups who are often those under-represented in PPIE research. We hope to be able extended the number of languages available in the future.

There are also limitations to this work. The AI field moves particularly quickly with the technical capabilities and use cases of LLMs constantly changing, which means that these animations may become dated. This is something that was apparent even during the co-design workshops, where different LLM products were becoming available, for example, AI Overview was a new feature Google launched during this period. We attempted to make these animations as future-proof as possible by focusing on the core aspects of how LLMs work, such as word prediction, and the training on a large dataset. Increasingly as AI literacy improves, there may also be interest in elements that we were unable to include in the limited time available in these animations such as the environmental impact of LLMs. Additionally some public contributors may find the animations simplistic and lacking technical detail depending on their prior knowledge and background.

In future work we would like to extend the number of languages that these animations are available in, to support a wider range of communities in LLM research in healthcare. Additionally, there are many avenues for future research to evaluate the impact of these animations on AI and LLM literacy.

As the application of LLMs in healthcare continues to grow, so will the need for public understanding of these technologies. These animated videos are a step towards ensuring that public and patient contributors have a say in how LLMs are used in healthcare and research.

## Data Availability

The original contributions presented in the study are included in the article/Supplementary Material, further inquiries can be directed to the corresponding author.
